# Spectroscopic Characterization and Cytotoxicity Assessment towards Human Colon Cancer Cell Lines of Acylated Cycloartane Glycosides from *Astragalus boeticus* L.

**DOI:** 10.3390/molecules24091725

**Published:** 2019-05-03

**Authors:** Vittoria Graziani, Assunta Esposito, Monica Scognamiglio, Angela Chambery, Rosita Russo, Fortunato Ciardiello, Teresa Troiani, Nicoletta Potenza, Antonio Fiorentino, Brigida D’Abrosca

**Affiliations:** 1 Dipartimento di Scienze e Tecnologie Ambientali Biologiche e Farmaceutiche (DiSTABiF), Università degli Studi della Campania “Luigi Vanvitelli”, via Vivaldi 43, I-81100 Caserta, Italy; vittoria.graziani@unicampania.it (V.G.); assunta.esposito@unicampania.it (A.E.); angela.chambery@unicampania.it (A.C.); rosita.russo@unicampania.it (R.R.); nicoletta.potenza@unicampania.it (N.P.); brigida.dabrosca@unicampania.it (B.D.A.); 2 Department of Biochemistry, Max Planck Institute for Chemical Ecology-Beutenberg Campus, Hans-Knöll-Straße, 8 D-07745 Jena, Germany; 3 Dipartimento di Medicina di Precisione, Università degli Studi della Campania “Luigi Vanvitelli” - Via Pansini, 5, 80131 Napoli, Italy; fortunato.ciardiello@unicampania.it (F.C.); teresa.troiani@unicampania.it (T.T.); 4 Dipartimento di Biotecnologia Marina, Stazione Zoologica Anton Dohrn, Villa Comunale, 80121 Naples, Italy

**Keywords:** *Astragalus boeticus* L., spectroscopic analysis, cytotoxic activity, human colon cancer cell lines, acetylated astragalosides, Fabaceae

## Abstract

In several European countries, especially in Sweden, the seeds of the species *Astragalus boeticus* L. were widely used as coffee substitutes during the 19th century. Nonetheless, data regarding the phytochemistry and the pharmacological properties of this species are currently extremely limited. Conversely, other species belonging to the *Astragalus* genus have already been extensively investigated, as they were used for millennia for treating various diseases, including cancer. The current work was addressed to characterize cycloartane glycosides from *A. boeticus*, and to evaluate their cytotoxicity towards human colorectal cancer (CRC) cell lines. The isolation of the metabolites was performed by using different chromatographic techniques, while their chemical structures were elucidated by nuclear magnetic resonance (NMR) (1D and 2D techniques) and electrospray-ionization quadrupole time-of-flight (ESI-QTOF) mass spectrometry. The cytotoxic assessment was performed in vitro by 3-(4,5-dimethylthiazol-2-yl)-2,5-diphenyltetrazolium bromide (MTT) assays in Caco-2, HT-29 and HCT-116 CRC cells. As a result, the targeted phytochemical study of *A. boeticus* enabled the isolation of three new cycloartane glycosides, 6-*O*-acetyl-3-*O*-(4-*O*-malonyl)-β-d-xylopyranosylcycloastragenol (**1**), 3-*O*-(4-O-malonyl)-β-d-xylopyranosylcycloastragenol (**2**), 6-*O*-acetyl-25-*O*-β-d-glucopyranosyl-3-*O*-β-d-xylopyranosylcycloastragenol (**3**) along with two known compounds, 6-*O*-acetyl-3-*O*-β-d-xylopyranosylcycloastragenol (**4**) and 3-*O*-β-d-xylopyranosylcycloastragenol (**5**). Importantly, this work demonstrated that the acetylated cycloartane glycosides **1** and **4** might preferentially inhibit cell growth in the CRC cell model resistant to epidermal growth factor receptor (EGFR) inhibitors.

## 1. Introduction

*Astragalus* genus is the largest in the Fabaceae family and it is widely distributed throughout the cool, temperate, semiarid and arid regions of the world [1]. *Astragalus boeticus* L. is a Steno-Mediterranean species, which has represented an important cultivation in several countries of Europe, as its seeds have been widely used as coffee substitutes in times of poverty and coffee prohibition. In Sweden, during the 19th century, the monarchy introduced an extensive cultivation of the aforementioned species to produce the so-called Swedish coffee. After the beginning of the 20th century, its cultivation declined, and it was replaced by other substitutes [2]. In addition to this information, available literature data describing the phytochemistry and the bioactivities of *A. boeticus* are currently extremely limited. 

On the contrary, a plethora of works regarding other species of the same genus exists. The *Astragalus* species were employed as forage for animals, albeit many species were found to be toxic, and responsible for causing locoism in cattle [3,4]. In both folk and modern medicine, several *Astragalus* spp. were considered medicinal plants of great importance, as these have been successfully used to cure a broad range of ailments [5]. In the Traditional Chinese Medicine “Astragali radix” (dried roots of *Astragalus membranaceus* Bunge and other *Astragalus* spp.) was a very well-known drug for its immune stimulant, hepato-protective, anti-diabetic, analgesic, expectorant and sedative properties [6]. 

Previous works investigated the chemical profile of *Astragalus* spp. in order to identify the active principles responsible for the bioactivity of the plant’s crude extracts. Results from these studies described imidazoline alkaloids, nitro toxins and selenium derivatives as toxic compounds, while polysaccharides, phenols and saponins as biologically active constituents [6]. *Astragalus* saponins include both oleanane and cycloartane-type glycosides, yet the former occur far less in nature, thus the *Astragalus* genus was especially employed as an ideal source to find cycloartane saponins [7]. 

These compounds were the most extensively studied secondary metabolites from *Astragalus*, as they exhibited a wide range of biological and pharmacological properties. Indeed, these molecules were found to exert immunomodulatory, anti-cancer, anti-fungal, hepato-, kidney-, neuro- and vascular-protective activities [7,8,9,10,11,12]. So far, the most well-characterized biological effects were those related to their immune stimulant properties, which made these compounds ideal vaccine adjuvant candidates [13]. Alongside the capacity to modulate key immunity pathways, recent evidence supported the effectiveness of *Astragalus* saponins as anti-tumor compounds and/or as adjuvants in combination with orthodox chemotherapeutic agents [14,15]. 

The anti-cancer activities of these compounds have been evaluated towards a wide range of human malignancies, and a large part of these works evidenced the effectiveness of *Astragalus* saponins against gastric and colorectal cancers [16]. Consistent with this, our group recently demonstrated the anti-proliferative effects of *A. boeticus* in human colorectal cancer (CRC) cells [17]. 

Colorectal cancer is one of the most frequently-diagnosed malignant diseases in Europe, and one of the leading causes of cancer-related deaths worldwide [18]. Even if the outcome of patients with metastatic colorectal cancer (mCRC) has clearly improved during the last years, the current therapies are still not entirely efficient. Nowadays, resistance to both chemotherapy and molecularly-targeted therapies represents a major problem for setting up effective treatment. The EGFR, which was found overexpressed in 60% to 80% of colorectal cancers, is a transmembrane tyrosine kinase receptor that, once activated, triggers two main signaling pathways. These include the RAS-RAF-MAPK axis, which is mainly involved in cell proliferation, and the PI3KPTEN-AKT pathway, which is especially involved in cell survival and motility [19]. Thus, EGFR inhibitors, such as Cetuximab and Panitumumab, have been developed to block specifically the abnormal activation of those pathways in wild-type KRAS CRC patients [20]. 

In this study, we aimed at providing a detailed chemical characterization of cycloartane glycosides from *A. boeticus*, and at assessing their anti-proliferative activity towards human colorectal cancer cells endowed with diverse mutation profiles and drug sensitiveness. The ultimate goal of this research is to contribute to the search for new effective agents against refractory CRCs. As a result, we isolated and characterized five cycloartane glycosides (**1**–**5**), identifying compound **4** as a strong inhibitor of proliferation in CRC cell models resistant to anti-EGFR therapies.

## 2. Results and Discussion

### 2.1. Structural Elucidation of Cycloartane Glycosides from Astragalus boeticus *L.*

**Figure 1:**
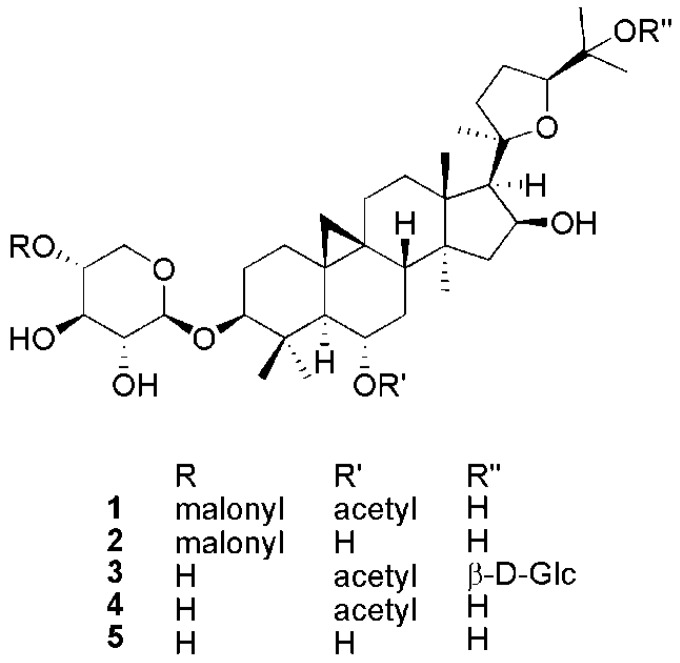
Structures of compounds **1**–**5**.

A crude hydro-alcoholic extract of *A. boeticus* leaves was partitioned between EtOAc and H_2_O. The purification process, which was performed by using different chromatographic techniques, enabled the isolation of compounds 1, 2, 4 from the organic phase, while we also obtained 3 and 5 from the aqueous fraction (Figure 1). The structures of these metabolites were elucidated through a combination of NMR spectroscopy (1D and 2D techniques) and ESI-QTOF mass spectrometry.

**Figure 2:**
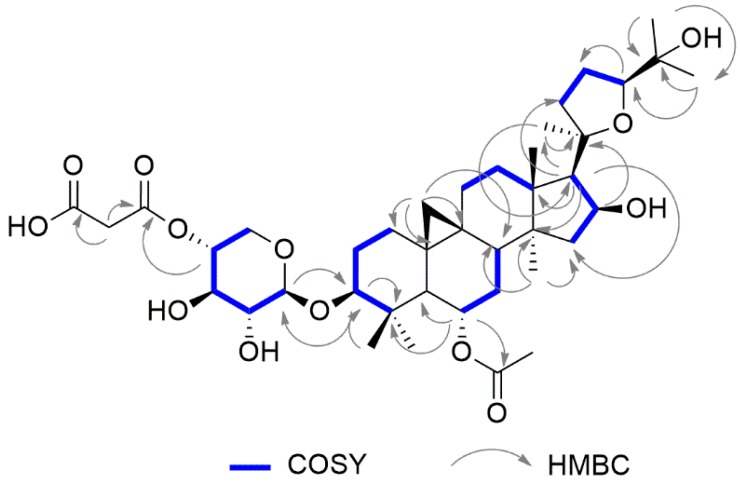
Selected H–H and H–C long range correlations of compound **1** evidenced in COSY and HMBC, respectively.

Compound **1** showed a molecular formula C_40_H_62_O_13_ on the basis of the NMR data and ESI-QTOF mass spectrum. In fact, the ^13^C NMR displayed 40 signals, which were identified using the HSQC experiment as eight methyls (—CH_3_), eleven methylenes (=CH_2_), eleven methines (=CH−), and ten quaternary carbons. The ESI-QTOF spectrum displayed the sodiated adduct of the quasimolecular ion at *m*/*z* 773.49, and a strong peak at *m*/*z* 687.46, which indicated the easy loss of an 86 Da fragment. In the ^1^H NMR spectrum (Table 1), two methylene protons at δ_H_ 0.40 and δ_H_ 0.61 (δ_C_ 30.1), along with six singlet methyls at δ_H_ 0.98, 1.01, 1.05, 1.13, 1.22, 1.26, and 1.27 allowed compound **1** to be identified as a cycloartane triterpene. The doublet at δ_H_ 4.32 (δ_C_ 105.9), as well as other protons that resonated in the range between 3.18 and 4.85 ppm, suggested the presence of a sugar unit. Meanwhile, a methyl singlet at δ_H_ 1.99 supported the presence of an acetate group in the molecule. The above-mentioned methylene signals (δ_H_ 0.40 and δ_H_ 0.61) were assigned to H-19 protons; these, in the CIGAR-HMBC experiment (Figure 2), showed cross peaks with the C-9 (δ_C_ 21.8), C-10 (δ_C_ 29.6), C-1 (δ_C_ 32.8) C-11 (δ_C_ 26.8), C-5 (δ_C_ 51.2) and C-8 (δ_C_ 46.7). In the same experiment, the H-5 proton correlated with the C-1 methylene, the C-4 quaternary carbon at δ_C_ 42.8, and with two carbinols at δ_C_ 89.1 and δ_C_ 72.0. Thanks to the long-range heterocorrelations between the H-6 proton (δ_H_ 4.75) and the C-4, C-5, and C-8 carbons, it was feasible to assign the first carbinol to the C-3 methine, and then the second to the C-6 carbon. Moreover, the H-6 proton also had cross peaks with the carbonyl at δ_C_ 171.7, which in turn correlated with the methyl at δ_H_ 1.99. These data allowed the acetoxy (CH_3_-COO-) group to be located at position 6. On the other hand, the H-3 proton correlated with the anomeric carbon at δ_C_ 105.9, suggesting that the glycosylation site was located on the hydroxyl at the C-3 carbon. Furthermore, the C-4 carbon showed correlations with the methyls at δ_H_ 0.98 and δ_H_ 1.05, values that were consequently assigned to the H-29 and H-28 protons, respectively. In addition, the methyls at δ_H_ 1.27 (δ_C_ 21.4) and 1.01 (δ_C_ 20.3) were attributed to the H-18 and H-30 protons, respectively, on the basis of the long range heterocorrelations between the C-18 carbon with the proton at δ_H_ 2.38 (H-17), which in turn correlated with the H-21 methyl at δH 1.22 (δ_C_ 28.4). 

In the COSY experiment, the H-17 proton had cross peaks with a proton geminal to oxygen at δH 4.65 (H-16), which homocorrelated with the methylene protons at δ_H_ 1.87 and δ_H_ 1.45 (H-15), suggesting the presence of another hydroxyl at the C-16 carbon. Consequently, the remaining methyls at δ_H_ 1.13 and δ_H_ 1.26 were attributed to the H-26 and H-27 protons, respectively. In the CIGAR-HMBC experiment, these latter protons displayed heterocorrelations with a quaternary carbinol carbon at δ_C_ 71.2 (C-25), and with a carbinol methine at δ_C_ 82.6, which was bound to a proton at δ_H_ 3.76. This latter signal evidenced heterocorrelations with the diasterotopic protons at δ_H_ 2.02 and δ_H_ 1.73, while homocorrelations were with the methylene protons at δ_H_ 2.62 and 1.87 (δ_C_ 35.5). All these data supported the presence of a tetrahydrofuran moiety formed by an oxygen bridge among the C-20 and C-24 carbons of the side chain. Besides the ester carbonyl of the acetate group, two additional carbonyls at δ_C_ 170.6 and 171.7 were also evident in the ^13^C NMR spectrum. In the CIGAR-HMBC experiment, both of them revealed cross peaks with the methylene at δ_H_ 3.69 (δ_H_ 51.8), while the carbon at δ_C_ 170.6 was further correlated with a proton at δ_C_ 4.72. These data were in agreement with the presence of a malonyl that was bound to the saccharide unit. In the HSQCTOXY experiment, the anomeric carbon at δ_C_ 105.9 showed cross peaks with the signals at δ_C_ 75.4, 75.2, 73.6 and δ_C_ 63.3. These carbons in the HSQC experiment correlated with the methines at δ_H_ 3.26, 3.57, 4.72 and to the methylene protons at δ_H_ 3.96 and 3.28, respectively. The H2BC experiment displayed the following correlations: Starting from the anomeric proton at δ_C_ 4.32 (H-1′)→75.4 (C-2′)→3.57 (H3′)→73.6 (C-4′)→3.96/3.28 (H-5′); starting from the anomeric carbon at δ_C_ 105.9 (C-1′)→3.26 (H-2′)→75.2 (C-3′)→4.72 (H-4′)→63.3 (C-5′). These data further supported the presence of a pentose that was bound to a malonyl group at the C-4′ carbon.

**Figure 3:**
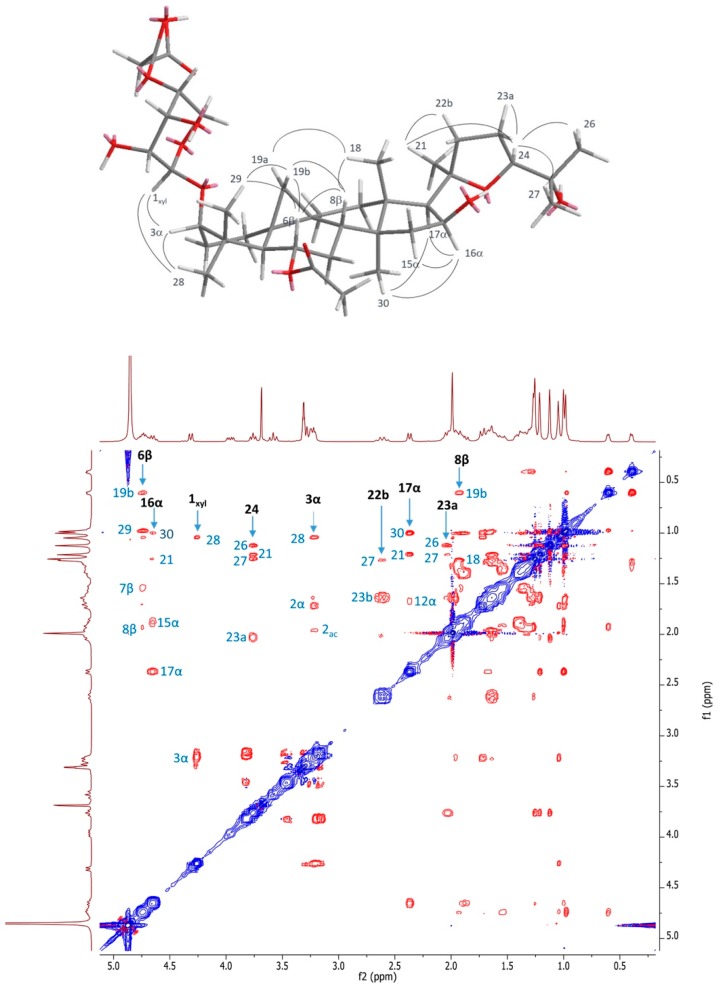
Key nOe correlation of compound **1** (top); NOESY experiment of compound **1** in CD_3_OD (bottom).

The stereostructure of the molecule was assigned on the basis of the nOe observed in the NOESY experiment (Figure 3). The sugar was identified as xylose by the GC-MS analysis of the acetylated alditol, which was obtained from the hydrolysis, reduction and acetylation of compound **1**. The coupling constant value of the anomeric proton allowed a β configuration for the anomeric carbon to be determined. The absolute configuration of the sugar was assigned by GC-MS, after a reaction of hydrolyzed saponins with l-cisteine methyl ester and acetylation [21]. 

The nOe evidenced in the NOESY experiments (Figure 3) allowed the configuration at the C-6 and C-16 carbons to be assigned. Based on these data, compound **1** was unequivocally identified as 6-*O*-acetyl-3-*O*-(4-*O*-malonyl)-β-d-xylopyranosylcycloastragenol (Figures S1–S9).

Compound **2** showed the molecular formula C_38_H_60_O_12_ on the basis of the NMR data, and the presence of a quasimolecular peak at *m*/*z* 731.41 in the ESI-QTOF MS spectrum. The loss of an 86 Da fragment was demonstrated by the peak at *m/z* 645.39, indicating the presence of a malonyl moiety also in this molecule. 

The ^1^H NMR spectrum showed the H-19 protons at δ_H_ 0.37 and 0.53, and seven singlet methyls at δ_H_ 0.96 (H-30), 0.98 (H-28), 1.15 (H-26), 1.24 (H-27), 1.26 (H-29 and H-21). The absence of the characteristic signal associated with the methyl group of the acetate moiety demonstrated that compound **2** was the deacetylated form of compound **1**, and all the NMR data confirmed this hypothesis. In addition, the C3-sugar was characterized as xylose, and the malonyl was positioned to the C-4′ carbon of this saccharide moiety. Based on these data, compound **2** was unequivocally identified as 3-*O*-β-d (4-*O*-malonyl)xylopyranosylcycloastragenol. 

Compound **3** showed the molecular formula C_43_H_70_O_15_, calculated on the basis of its spectro-scopic features. In the ^1^H-NMR spectrum, the signals of the aglycone, and those belonging to the C3-xylopyranose, were in good agreement with the previous metabolite. However, a further anomeric doublet at δ_C_ 4.51 suggested the presence of a second sugar. The heterocorrelation between this anomeric signal and the C-25 carbon at δ_H_ 79.9 allowed the second site of glycosylation to be located at position 25. The HSQCTOCSY experiment revealed the presence of an additional spin system due to this sugar, in which the anomeric proton (δ_C_ 4.51) correlated with the carbons at δc 99.6, 75.0, 78.2, 71.2, 77.5 and 62.7. These data were in agreement with the presence of a glucopyranose, a hypothesis that was confirmed by GC-MS analysis. Moreover, the coupling constant value indicated a β configuration for the anomeric carbon, while the D-series was established by GC-MS after the reaction of hydrolyzed saponins with L-cisteine methyl ester and acetylation. Based on these data, compound 3 was unequivocally identified as 6-*O*-acetyl-25-*O*-β-d-glucopyranosyl-3-*O*-β-d-xylo-pyranosylcycloastragenol.

Compound **4** showed a molecular formula C_37_H_60_O_10_, calculated on the basis of its spectroscopic data. Of interest, this compound was also obtained by the mild acidic hydrolysis of compound **1**. In fact, when this compound was dissolved in water, after one day at room temperature, it was quantitatively converted in **4** by loss of the C4′-malonyl. All the NMR data confirmed this hypothesis, and allowed the identification of compound **4** as 6-*O*-acetyl-3-*O*-β-d-xylopyranosylcycloastragenol [22]. 

Compound **5** showed a molecular formula C_35_H_58_O_9_, calculated on the basis of its spectroscopic data. The ^1^H-NMR and ^13^C-NMR signals of the aglycone and those belonging to the C3-xylopyranose were superimposable with the previous metabolites. Conversely, the lack of the singlet peak at δ_H_ 1.99 proved the absence of the C6-acetyl. All of these data enabled the identification of compound **5** as 3-*O*-β-d-xylopyranosylcycloastragenol [23].

### 2.2. Cytotoxicity of Cycloartane Glycosides from Astragalus boeticus Against Human Colorectal Cancer Cells

**Figure 4:**
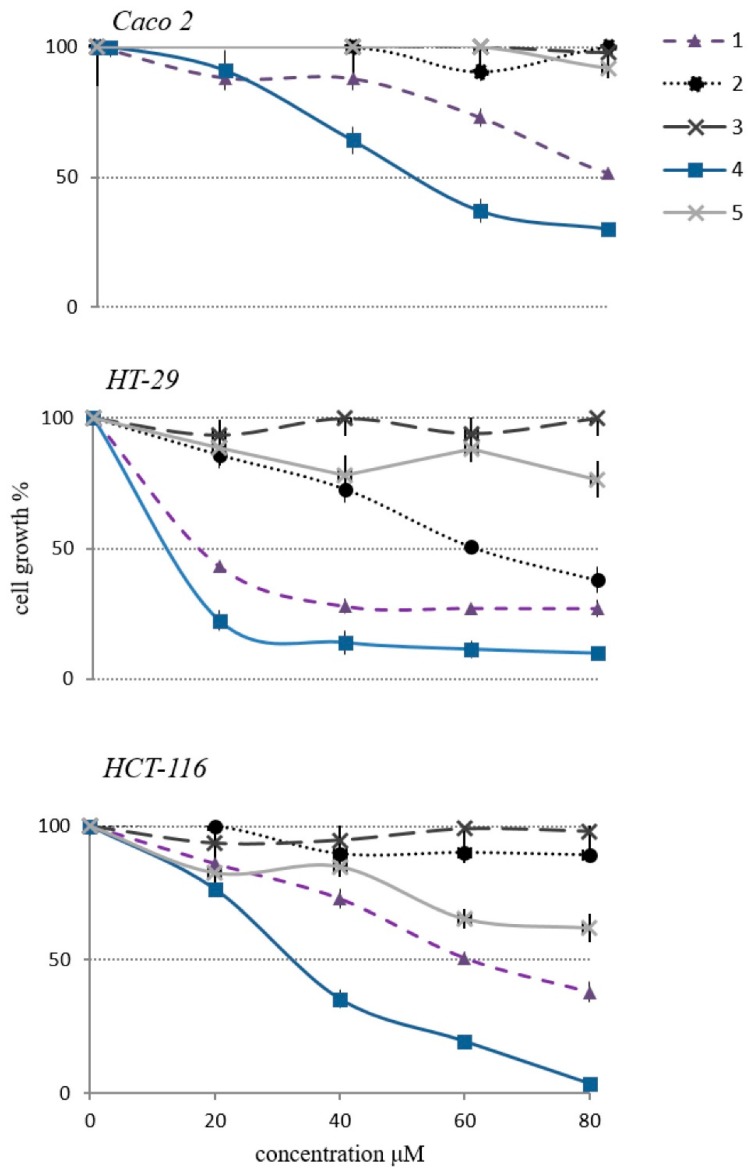
Cytotoxic activity of compounds **1**–**5** towards Caco-2, HT-29 and HCT-116 human colon cancer cell lines.

The cytotoxic activity of the isolated compounds (**1**–**5**) was assessed on three human colorectal cancer cell lines (Caco-2, HT-29 and HCT-116), using MTT (3-[4,5-dimethylthiazol-2-yl]-2,5-diphenyltetrazolium bromide) tetrazolium salt colorimetric assay (Figure 4). Results from these experiments demonstrated that cell proliferation was reduced by the treatment with compounds **1** and **4** in a dose-dependent manner, while **2**, **3** and **5** did not exert any significant effect. To our knowledge, no mechanistic studies regarding the proliferation reducing-effect of compounds **1** and **4** are available in literature. Yet, several previous works shed the light on the anticancer activity of other cycloartane glycosides (most of them isolated from *A. membranaceus*), which act by inducing apoptosis and modulating crucial cellular signaling pathways [24,25,26,27]. Importantly, a recent investigation pointed out that certain semisynthetic cycloastragenol derivatives impair inflammation-carcinogenesis by regulating the NF-KB signaling pathway [15]. 

Consistently, compound **4**, which has already been purified from the leaf extract of *A. membranaceus*, was described as a potentially anti-inflammatory molecule, because it was found to exert an inhibitory activity on the nitric oxide production in macrophages [21]. Recently, our group identified compound **4** as a metabolite responsible for the cytotoxicity of the *A. boeticus* extract. Here, this species underwent a further phytochemical investigation to understand whether analogues of compound **4** exert cytotoxicity as well. As it was already anticipated above, we demonstrated that compounds **1** and **4** were the active cycloartane glycosides isolated from *A. boeticus*. 

In an attempt to further validate our findings, we compared the cytotoxicity of **4** (active compound) and **3** (inactive compound) with astragaloside IV (AS-IV), a commercially-available cycloartane glycoside extensively recognized as one of the main active components of several *Astragalus* spp. Results from these experiments confirmed the notable cytotoxicity of compound **4**, while no effect was found for AS-IV, at least in our experimental conditions (Figure S10). 

From a structural point of view, these insights proved that the C3-xylopyranosyl, along with the C6-acetoxy group and the C25-free hydroxyl function, were essential structural requirements for the cytotoxic activity of these triterpenoids. As already discussed, when compound **1** was in water solutions, such as a cellular environment, it converted into compound **4** by losing the C4’ malonyl. Thereby, it was demonstrated that compound **4** was the real bioactive structure. Compounds **2**, **3**, **5** and AS-IV present the C3-xylopyranosyl, while none of them has either the C6-acetoxy group or the C25-free hydroxyl function. In accordance with previous investigations, acylation of the C-3 and C-6 secondary alcohols of diverse cycloartane derivatives resulted in a higher cytotoxic activity. This evidence led researchers to hypotheses that acylated cycloartane glycosides could lose the acyl substituents to modify proteins that play a key role in cellular signaling, whose deregulation is involved in carcinogenesis. Specifically, the acyl groups can be covalently attached to the amino acid side chains regulating the protein functions and impairing cancer progression [15]. 

In our study, a detailed analysis of the cytotoxic effect unveiled a differential response of Caco-2, HT-29 and HCT-116 to the treatment with compound **4**. These insights could be interpreted by describing the human colon cancer cells employed in our experimental setting. Caco-2 cell line was the wild type for the KRAS, NRAS, BRAF, PIK3CA genes; thus representing an ideal cellular model to study metastatic CRCs sensitive to anti-EGFR agents. By contrast, HT-29 and HCT-116 harbored a BRAF and some KRAS/PIK3CA mutations, respectively—therefore identifying metastatic CRCs with intrinsic resistance to the anti-EGFR treatments [28,29]. 

Herein, the in vitro cytotoxic screening revealed as compound **4** was clearly more effective in treating HT-29 (3 µM) than HCT-116 (40 µM) and Caco-2 (50 µM) cell lines (Figure 4).

In clinics, the BRAF mutation status is a strong indicator of a very poor prognosis for mCRC patients; indeed, they showed a worse outcome for the therapies compared with those whose tumors were wild type [30,31,32]. Of interest, the inhibition of BRAF oncoprotein by the small-molecule drug PLX4032 (Vemurafenib) is highly effective in the treatment of melanoma [33], whilst metastatic CRC patients associated with the same mutation showed a very limited response to this drug [34]. In fact, Vemurafenib treatment induces EGFR feedback activation, causing continued proliferation in the presence of BRAF (V600E) inhibition. Unlike CRCs, melanomas express low levels of EGFR, and thus, they are not subject to this kind of feedback activation [35].

## 3. Materials and Methods 

### 3.1. General Experimental Procedures

Analytical TLC was performed on Merck Kieselgel (Darmstadt, Germany) 60 F254 or RP-8 F254 plates with a 0.2 mm film thickness. Spots were visualized by UV light, or by spraying with H_2_SO_4_/AcOH/H_2_O (1:20:4), and then heating at 120 °C for 5 min. Preparative TLC was performed on Merck Kieselgel 60 F254 plates with a 0.5 or 1.0 mm film thickness. 

Column chromatography (CC) was performed on Fluka (Seelze, Germany) Amberlite XAD-4 and XAD-7, on Pharmacia (Stockholm, Sweden) Sephadex LH-20, on Merck Kieselgel 60 (70–240 mesh), or on Baker (Deventer, Netherlands) RP-8. Nuclear magnetic resonance (NMR) spectra were recorded at 300 (^1^H) and 75 MHz (^13^C) on a Varian Mercury 300 FT-NMR spectrometer in CD_3_OD or pyridine-d_5_ solutions at 25 °C. Chemical shifts are reported in δ (ppm), and referenced to the residual solvent signal; J (coupling constant) are given in Hz. Standard pulse sequences and phase cycling from Varian library were used for ^1^H, ^13^C, DEPT, DQF-COSY, COSY, TOCSY, NOESY, HSQC, H2BC, HMBC and CIGAR–HMBC experiments. ^1^H NMR spectra were acquired over a spectral window from 14 to −2 ppm, with 1.0 s relaxation delay, 1.70 s acquisition time (AQ), and 90° pulse width = 13.8 μs. The initial matrix was zero-filled to 64 K. ^13^C NMR spectra were recorded in ^1^H broadband decoupling mode, over a spectral window from 235 to −15 ppm, 1.5 s relaxation delay, 90° pulse width = 9.50 μs, and AQ = 0.9 s. The number of scans for both ^1^H and ^13^C NMR experiments were chosen, depending on the concentration of the samples. With regards to the homonuclear and heteronuclear 2D-NMR experiments, the data points, number of scans and increments were adjusted according to the sample concentrations. Correlation spectroscopy (COSY) and double quantum filtered COSY (DQF-COSY) spectra were recorded with gradient-enhanced sequence at spectral widths of 3000 Hz in both f2 and f1 domains; the relaxation delays were of 1.0 s. The total correlation spectroscopy (TOCSY) experiments were performed in the phase-sensitive mode with a mixing time of 90 ms. The spectral width was 3000 Hz. Nuclear Overhauser effect spectroscopy (NOESY) experiments were performed in the phase-sensitive mode. The mixing time was 500 ms, and the spectral width was 3000 Hz. For all the homonuclear experiments, the initial matrix of 512 × 512 data points was zero-filled to give a final matrix of 1 k × 1 k points. Proton-detected heteronuclear correlations were also measured. Heteronuclear single-quantum coherence (HSQC) experiments (optimized for 1*J*(H,C) = 140 Hz) were performed in the phase sensitive mode with field gradient. The spectral width was 12,000 Hz in f1 (^13^C) and 3000 Hz in f2 (1H) and 1.0 s of relaxation delay; the matrix of 1 k × 1 k data points was zero-filled to give a final matrix of 2 k × 2 k points. Heteronuclear 2 bond correlation (H2BC) spectra were obtained with T = 30.0 ms, and a relaxation delay of 1.0 s; the third order low-pass filter was set for 130 < 1*J*(C,H) < 165 Hz. A heteronuclear multiple bond coherence (HMBC) experiment (optimized for 1*J*(H,C) = 8 Hz) was performed in the absolute value mode with field gradient; typically, ^1^H–^13^C gHMBC were acquired with spectral width of 18,000 Hz in f1 (^13^C) and 3000 Hz in f2 (1H) and 1.0 s of relaxation delay; the matrix of 1 k × 1 k data points was zero-filled to give a final matrix of 4 k × 4 k points. Constant time inverse-detected gradient accordion rescaled heteronuclear multiple bond correlation spectroscopy (CIGAR–HMBC) spectra (8 > n*J*(H,C) > 5) were acquired with the same spectral width used for HMBC. Heteronuclear single quantum coherence-total correlation spectroscopy (HSQC-TOCSY) experiments were optimized for n*J*(H,C) = 8 Hz, with a mixing time of 90 ms. For accurate mass measurements, the purified compounds were analyzed by an electrospray hybrid quadrupole orthogonal acceleration time-of-flight mass spectrometer (Q-TOF), fitted with a Z-spray electrospray ion source (Waters S.p.A.). All analyses were carried out in positive ion mode. The capillary source voltage and the cone voltage were set at 3500 V and 35 V, respectively. The source temperature was kept at 80 °C, and nitrogen was used as a drying gas (flow rate about 50 l/h). The time-of-flight analyzer of the mass spectrometer was externally calibrated, with GFP from *m*/*z* 50–1600. Accurate mass data were collected by directly infusing samples (1.5 pmol/μL in CH_3_CN/H_2_O, 1:1) into the system at a flow rate of 15 μL/min. The acquisition and processing of data were performed with the MassLynx 4.1 software (Waters S.p.A., Manchester, UK). GC-MS analyses were carried out using an HP 6890 GC instrument (Zebron ZB-5MS column, He flow 1.0 mL/min), coupled with a 5973 N mass spectrometer, equipped with an electron ionization source (EIMS), and operating with an electron energy of 70 eV. Full-scan mass spectra were collected between 0 and 600 amu at 2 scan/s. The MS was operated in the electron impact (EI) ionization mode, with an electron energy of 70 eV. The ion source and quadrupole temperatures were maintained at 230 and 150 °C, respectively. For the analyses of the acetylated alditols, the column head pressure was set at 7.41 p.s.i. 

AS-IV was purchased from Shanghai Tauto Biotech Co., LTD. (Shanghai, China).

### 3.2. GC–MS Analysis of the Sugar Moieties

The GC-MS analysis of the sugar moieties has been previously described by Scognamiglio et al. [31]. Briefly, each metabolite (0.5 mg) was subjected to an acid hydrolysis with 2 N TFA (150 µL) at 120 °C for 1 h, obtaining the sugar moiety. This was dried under N2 flow, and reduced by adding MeOH (150 µL) and NaBH_4_ (1.0 mg). The solution was incubated at room temperature for 1 h and then dried under N_2_ flow after treatment with glacial AcOH and MeOH. The obtained alditol was acetylated by using anhydrous pyridine (200 µL) and Ac_2_O (200 µL). This mixture was incubated for 20 min at 120 °C. Then, 500 µL of H_2_O was added, and the product was extracted with CH_2_Cl_2_ (500 µL) following centrifugation at 3500 rpm for 5 min. The organic phase was dried under N_2_ flow, dissolved in CH_2_Cl_2_ (500 µL) and analyzed by GC-MS. Temperature conditions were as follows: Injector port at 250 °C; the initial oven temperature was 160 °C for 50 s, then linearly increased to 200 °C at 10 °C/min. A further linear increase at 2.5 C/min was performed to 300 °C, and held for 40 min. Sample solutions were injected using the split mode.

### 3.3. Determination of Absolute Configuration of Monosaccharides of Compound ***1*** and ***5***

Compound **1** and **5** (2 mg each) were hydrolyzed with 2 N TFA (250 µL) at 120 °C for 1 h. The reaction mixture was then dried under N_2_ flow and dissolved in dry pyridine (100 µL). 100 µL of pyridine solution of l-cysteine methyl ester hydrochloride (0.06 mol/L) were added to pyridine solutions of the hydrolyzed compounds **1** and **5** and pure d-xylose, and l-xylose (0.04 mol/L). These mixtures were warmed at 60 °C for 1 h, afterwards, acetic anhydride (150 µL) was added at 120 °C for 20 min. The products were dried under N_2_, dissolved in 500 µL of H_2_O, and extracted with CH_2_Cl_2_ (500 µL) following centrifugation at 3500 rpm for 5 min. The organic phase was dried under N_2_ flow, dissolved in CH_2_Cl_2_ (500 µL), and analyzed by GC-MS. Temperature conditions were as follows: Injector port at 250 °C; initial oven temperature 45 °C, then increased linearly to 300 °C at 20 °C/min, and then held for 25 min. Sample solutions were injected using the split mode. The retention times were: d-xylose 13.89 min, l-xylose 14.17 min.

### 3.4. Plant Material

Leaf samples of *A. boeticus* were collected at vegetative state, in April 2014 in “Castel Volturno Nature Reserve” (40°57.587′N, 14°00.105′E; southern Italy). Samples were harvested, frozen in liquid nitrogen and lyophilized. A Voucher specimen CE000016 has been deposited at the Herbarium of the Dipartimento di Scienze e Tecnologie Ambientali Biologiche e Farmaceutiche of Università degli Studi della Campania “Luigi Vanvitelli”.

### 3.5. Extraction and Isolation of Compound ***1***–***5***

Dried leaves (24.0 g) of *A. boeticus* were powdered, and underwent three cycles of an ultrasound-assisted extraction with an MeOH/H_2_O (1:1) solution (720 mL) [36], finally obtaining a crude extract (7.1 g). This was dissolved in H_2_O and separated by liquid–liquid extraction, by using EtOAc as our extracting solvent. As a result, an organic and a water fraction were obtained. The former (1g) was chromatographed by SiO_2_ CC, and eluted using a solution with an increasing degree of polarity (CHCl_3_, Me_2_CO/CHCl_3_, MeOH/CHCl_3_). Thus, 21 fractions have been collected. Of these, number 13 was chromatographed by C18 CC, and eluted with H_2_O/MeOH (3:2) to give compound **1** (18.6 mg) and 2 (7.1 mg). Meanwhile, number 12 was purified by Flash-CC eluting with MeOH/ CHCl_3_ (3:100) to obtain compound 4 (28 mg). On the other hand, the water fraction was chromatographed by XAD-4 (20–50 mesh; Fluka) and XAD-7 (20–50 mesh; Fluka) CC, obtaining an alcoholic eluate (900 mg) that was in turn purified by Sephadex LH-20. Subsequently, 17 fractions were given. Of these, fraction 13 was purified through RP-18 CC by using H_2_O/MeOH (4:1) as the eluting system, consequently obtaining 30 fractions. One of these was chromatographed by TLC (0,5 mm), and eluted with the organic phase of CHCl_3_/MeOH/H_2_O (13:7:2) to obtain compound **3** (11.8 mg). Finally, fraction 11 was chromatographed by TLC (0.5 mm) and eluted with the organic phase of CHCl_3_/MeOH/H_2_O (13:7:2) to give compound **5** (4.6 mg). 

Compound **1**: 6-*O*-acetyl-3-*O*-(4-*O*-malonyl)-β-d-xylopyranosylcycloastragenol. [α]_D_^25^ = +22.8 (c = 14.97 × 10^−3^, MeOH). ^1^H NMR (CD_3_OD) and ^13^C NMR (CD_3_OD) see Table 1; ESI/Q-TOF: *m*/*z* 773.49 [M + Na]^+^ (calcd.773.41 Da for C_40_H_62_O_13_Na); 

Compound **2**: 3-*O*-(4-*O*-malonyl)-β-d- xylopyranosylcycloastragenol. [α]_D_^25^ = +9.70 (c = 2.06 × 10^−3^, MeOH). ^1^H NMR (CD_3_OD) and ^13^C NMR (CD_3_OD) see Table 1; ESI/Q-TOF: *m/z* 731.41 [M+Na]+ (calcd. 731.40 Da for C_38_H_60_O_12_Na);

Compound **3**: 6-*O*-acetyl-25-*O*-β-d-glucopyranosyl-3-*O*-β-d-xylopyranosylcycloastragenol. [α]_D_^25^ = +13.7 (c= 6.06 × 10^−3^, MeOH). ^1^H NMR (CD_3_OD) and ^13^C NMR (CD_3_OD) see Table 1; ESI/Q-TOF: *m*/*z* 849.46 [M + Na]^+^ (calcd.849.43 Da for C_43_H_70_O_15_Na);

Compound **4**: 6-acetyl-3-*O*-(4-*O*-malonyl)-β-d-xylopyranosylcycloastragenol. [α]_D_^25^ = +7.65 (c = 5.1 × 10^−3^, MeOH/H_2_O, 2:1) [22];

Compound **5**: 3-*O*-β-D-xylopyranosylcycloastragenol. [α]_D_^25^ = −340.1 (c = 3.74 × 10^−3^) MeOH/H_2_O, 1:1) [23].

### 3.6. Cell Lines 

The human HCT-116, HT-29, Caco-2 colorectal cancer cell lines were obtained from the American Type Culture Collection (ATCC) (Manassas, VA). HCT-116, HT-29 cancer cells were cultured in RPMI 1640 medium (Lonza, Cologne, Germany) supplemented with 10% fetal bovine serum, 2 mM l-glutamine, 50 U/mL penicillin and 100 µg/mL streptomycin (Lonza, Cologne, Germany). The Caco-2 cell line was cultured in DMEM medium (Lonza, Cologne, Germany), supplemented with 10% fetal bovine serum, 2 mM l-glutamine, 1% non-essential amino acid, 50 U/mL penicillin and 100 µg/mL streptomycin (Lonza, Cologne, Germany). 

### 3.7. Proliferation Assay

The cell proliferation assay was performed with a 3-(4,5-dimethylthiazol-2-yl)-2,5-diphenyl-tetrazolium bromide (MTT) assay. Briefly, cells in logarithmic growth phase were plated in 96-well plates and incubated for 24 h before exposure to increasing doses of DMSO-diluted compounds (20, 40, 60, and 80 µM). For compounds **1** and **4** that exerted a strong cytotoxic effect in the HT-29 cell line, a lower range of doses (2, 4, 6, and 10 µM) was also investigated. 48 h after treatment, 50 µL of 1 mg/mL (MTT) were mixed with 200 μL of medium and added to the well. 1 h after incubation at 37 °C, the medium was removed, and the purple formazan crystals produced in the viable cells were solubilized in 100 μL of dimethyl sulfoxide, and quantitated by measurement of absorbance at 570 nm with a plate reader. Results were reported as mean ± s.d. of % of cell growth, with respect to the control from six replicate analyses. The control was represented by a 0.08% DMSO treatment, which corresponded to the higher amount of DMSO used for the tests.

## 4. Conclusions

A plethora of previous investigation regarding the phytochemistry and the bioactive components of diverse species belonging to the *Astragalus* genus are available in literature. However, to our knowledge, there are no data regarding the phytochemical constituents of *A. boeticus*. In this study, we focused on the cycloartane derivatives present in *A. boeticus*, because of its putative cytotoxic activity. 

Specifically, the targeted phytochemical study of *A. boeticus* led to the isolation of five cycloartane-type glycosides (**1**–**5**); of these, **1**, **2** and **3** were isolated and characterized for the first time. The cytotoxic activity of compounds **1**–**5** was evaluated in vitro, disclosing a strong proliferation-reducing effect of compound **4** in the HT-29 human colon cancer cell line, which was employed as a preclinical model to study refractory mCRCs that are resistant to anti-EGFR therapies, as well as other chemotherapeutic drugs currently used in the clinical setting. Our results therefore provide a small molecule scaffold that might be potentially important for the development of new agents effective against refractory mCRCs.

Indeed, these insights pave the way to further investigations aimed to figure out whether compound **4** acts on CRC cell models resistant to EGFR inhibitors with high selectivity, and if so, to elucidate the mechanism by which the anti-proliferative activity occurs.

On the other hand, as several limitations are intrinsically associated with our in vitro experimental system, future experiments shall also be addressed to evaluate the pharmacokinetic properties, and the bioavailability of the active molecule in animal models.

## Figures and Tables

**Table 1:** 1D and 2D nuclear magnetic resonance (NMR) data of compound **1**–**3** in CD_3_OD.

	1				2				3			
Position	δ_C_	Type	δ_H_ (*J* in Hz)	HMBC^a^	δ_C,_	Type	δ_H_ (*J* in Hz)	HMBC ^a^	δ_C,_	Type	δ_H_ (*J* in Hz)	HMBC ^a^
1	32.8	CH_2_	1.85 *s* 1.67 *s*	2, 10 2, 10	32.3	CH_2_	1.84 *s* 1.65 *s*	2, 10 2, 10	32.8	CH_2_	1.29 s	
2	30.3	CH_2_	1.98 *ov* 1.64 *ov*	3, 4 3, 4	30.0	CH_2_	1.88 *ov* 1.69 *ov*	3, 4 3, 4	30.2	CH_2_	1.99 ov	3, 4
3	89.1	CH	3.21 *d* (*J* = 1.8)	28, 29, 1_xyl_	89.1	CH	3.29 *ov*	28, 29, 1_xyl_	89.1	CH	3.21 *ov*	1_xyl_
4	42.8	C			42.0	C			42.8	C		
5	51.2	CH	1.71 *s*	1, 4, 7, 10, 28, 29	53.9	CH	1.38 *s*	1, 4, 7, 10, 28, 29	51.3	CH	1.72 *s*	1, 4, 7, 10, 28, 29
6	72.0	CH	4.75 *ov*	4, 5, 7, 8, 1_Ac_	68.5	CH	3.48 *ov*	4, 5, 7, 8	72.1	CH	4.75 *ov*	4, 5, 1_ac_
7	34.0	CH_2_	1.56 *ov*	5, 6	33.4	CH_2_	1.46 *ov*	5, 6	34.1	CH_2_	1.57	5, 6, 8, 9, 14
8	46.7	CH	1.95 *s*	6, 9, 10, 13, 14, 19, 30	48.0	CH	1.46 *s*	6, 9, 10, 13, 14, 19, 30	46.1	CH	1.94 *s*	6, 10, 13, 14, 15, 19
9	21.8	C			21.4	C			21.6	C		
10	29.6	C			29.2	C			29.2	C		
11	26.8	CH_2_	1.97 *m*	8, 12, 19	26.4	CH_2_	1.97 *s*	8, 12, 19	26.4	CH_2_	1.97 *s*	12
12	34.1	CH_2_	1.64 *ov* 1.56 *ov*	14, 18 14, 18	33.4	CH_2_	1.54 *ov* 1.37 *ov*	14, 18 14, 18	34.1	CH_2_	1.64 *ov* 1.56 *ov*	
13	47.0	C			44.7	C			46.9	C		
14	46.4	C			47.0	C			46.6	C		
15	46.2	CH_2_	1.87 *d* (*J* = 8.0) 1.39 *d* (*J* = 8.0)	8, 13, 17, 30 8, 13, 17, 30	46.4	CH_2_	1.98 *d* (*J* = 4.4) 1.32 *d* (*J* = 6.8)	8, 13, 17, 30 8, 13, 17, 30	46.0	CH_2_	1.86 *d* (*J* = 6.0) 1.42 *d* (*J* = 6.4)	8, 13, 14, 17, 30 13, 14, 30
16	74.5	CH	4.65 *m*	13, 14, 15	73.9	CH	4.64 *m*	13, 14, 15	74.3	CH	4.65 *m*	13, 14, 15
17	58.9	CH	2.37 *d* (*J* = 7.8)	13, 14, 16, 20, 21, 22	58.2	CH	2.37 *d* (*J* = 7.7)	13, 14, 16, 20, 21, 22	58.7	CH	2.38 *d* (J=8.0)	13, 14, 16, 20, 21, 22
18	21.4	CH_3_	1.27 *s*	12, 15, 17	21.4	CH_3_	1.23 *s*	12, 15, 17	21.7	CH_3_	1.27 *s*	12, 15, 17
19	30.1	CH_2_	0.61 *s* 0.40 *s*	1, 5, 8, 9, 10 1, 5, 8, 9, 10, 11	31.4	CH_2_	0.53 *s* 0.37 *s*	1, 5, 8, 9, 10 1, 5, 8, 9, 10, 11	30.2	CH_2_	0.61 *s* 0.40 *s*	1, 5, 8, 9, 10,11 1, 5, 8, 9, 10, 11
20	88.3	C	-		88.2	C	-		88.3	C	-	
21	28.4	CH_3_	1.22 *s*	17, 20, 22	28.1	CH_3_	1.25 *s*	17, 20, 22	28.0	CH_3_	1.24 *s*	
22	35.5	CH_2_	2.62 *ov* 1.87 ov	17, 20, 21 17, 20, 21	34.9	CH_2_	1.69 *ov*	17, 20, 21 17, 20, 21	35.6	CH_2_	2.54 *ov* 1.84	17 17
23	26.8	CH_2_	2.02 *ov* 1.73	20, 24, 25 17, 24, 25	26.4	CH_2_	2.02 *ov*	20, 24, 25 17, 24, 25	26.8	CH_2_	2.16 *ov* 1.72	24, 25 24, 25
24	82.6	CH	3.76 *m*	25	81.4	CH	3.80 *m*	25	83.0	CH	3.82 *m*	1_glc_
25	71.2	C	-		71.2	C	-		79.9	C	-	
26	26.6	CH_3_	1.13 *s*	17, 24, 25	26.5	CH_3_	1.15 *s*	17, 24, 25	23.2	CH_3_	1.22 *s*	20, 23, 24, 25
27	27.6	CH_3_	1.26 *s*	24, 25, 26	27.1	CH_3_	1.24 *s*	24, 25, 26	25.3	CH_3_	1.38 *s*	23, 24, 26
28	27.2	CH_3_	1.05 *s*	3, 4, 5, 29	16.1	CH_3_	0.98 *s*	3, 4, 5, 29	16.5	CH_3_	0.98 *s*	3, 4, 5, 28
29	16.6	CH_3_	0.98 *s*	3, 4, 5, 28	27.1	CH_3_	1.25 *s*	3, 4, 5, 28	27.3	CH_3_	1.04 *s*	3, 4, 5, 29
30	20.3	CH_3_	1.01 *s*	9, 14, 15	20.9	CH_3_	0.96 *s*	9, 14, 15	20.2	CH_3_	0.99 *s*	14, 15
1_xyl_	105.9	CH	4.32 *d* (*J* = 7.4)	3, 5_xyl_	105.9	CH	4.43 *d* (*J* = 7.4)	3, 5_xyl_	107.4	CH	4.26 *d* (*J* = 7.0)	3
2_xyl_	75.4	CH	3.26 *ov*	4_xyl_	75.4	CH	3.26 *ov*	4_xyl_	75.5	CH	3.19 *ov*	
3_xyl_	75.2	CH	3.57 *ov*	4_xyl_, 5_xyl_	75.2	CH	3.57 *ov*	4_xyl_, 5_xyl_	78.0	CH	3.29 *ov*	
4_xyl_	73.6	CH	4.72 *m*	2_xyl_, 3_xyl_, 5_xyl_, 2_mal_	73.6	CH	4.72 *m*	2_xyl_, 3_xyl_, 5_xyl_, 2_mal_	71.2	CH	3.46 *m*	
5_xyl_	63.3	CH_2_	3.28 *ov* 3.96 *ov*	1_xyl_, 3_xyl_, 4_xyl_ 1_xyl_, 3_xyl_, 4_xyl_	63.3	CH_2_	3.28 *ov* 3.96 *ov*	1_xyl_, 3_xyl_, 4_xyl_ 1_xyl_, 3_xyl_, 4_xyl_	66.7	CH_2_	3.18 *ov* 3.82 *ov*	
1_glc_	-	-	-		-		-		99.6	CH	4.51 *d* (*J* = 6.6)	25
2_glc_	-	-	-		-		-		75.0	CH	3.18 *ov*	5_glc_
3_glc_	-	-	-		-		-		78.2	CH	3.32 *ov*	1_glc_, 5_glc_
4_glc_	-	-	-		-		-		71.2	CH	3.31 *ov*	6_glc_
5_glc_	-	-	-		-		-		77.5	CH	3.24 *ov*	1_glc_, 3_glc_
6_glc_	-	-	-		-		-		62.7	CH_2_	3.65 *ov* 3.79 *ov*	4_glc_ 4_glc_
1_ac_	172.2	C	-		-		-		172.2	C		
2_ac_	22.2	CH_3_	1.99 *s*	1_ac_	-		-		21.8	CH_3_	1.99 *s*	1_ac_
1_mal_	170.6	C	-		170.6	C	-		-		-	
2 _mal_	51.8	CH_2_	3.69 *s*	1_mal,_ 3_mal_	51.8	CH_2_	3.69 *s*	1_mal,_ 3_mal_	-		-	
3 _mal_	171.7	C	-		171.7	C	-		-		-	

^a^ HMBC correlations, optimized for 6 Hz, are from proton(s) stated to the indicated carbon; ^b^ obscured; *d* = doublet, *m* = multiplet, *ov* = overlapped, *s* = singlet, *t* = triplet.

## Supplementary Materials

The following are available online at https://www.mdpi.com/1420-3049/24/9/1725/s1, Figure S1: ^1^H NMR spectrum of compound **1**. Figure S2: ^13^C NMR spectrum of compound **1**. Figure S3: COSY spectrum of compound **1**. Figure S4: HSQC spectrum of compound **1**. Figure S5: CIGAR-HMBC spectrum of compound **1**. Figure S6: H2BC spectrum of compound **1**. Figure S7: HSQCTOCSY spectrum of compound **1**. Figure S8: ESI QTOF spectrum of compound **1**. Figure S9: ESI TOF MS/MS spectrum of compound **1**. Figure S10: cytotoxicity of AS-IV in comparison with compound **3** and **4**.

## References

[1] Verotta L., El-Sebakhy N. (2001). Cycloartane and oleanane saponins from Astragalus sp. Studies in Natural Products Chemistry.

[2] Prohens J., Andújar I., Vilanova S., Plazas M., Gramazio P., Prohens R., Herraiz F.J., De Ron A.M. (2013). Swedish coffee (Astragalus boeticus L.), a neglected coffee substitute with a past and a potential future. Genet. Resour. Crop Evol..

[3] Williams M.C., Davis A.M. (1982). Nitro Compounds in Introduced Astragalus Species. J. Range Manage..

[4] Cook D., Ralphs M.H., Welch K.D., Stegelmeier B.L. (2009). Locoweed Poisoning in Livestock. Rangelands.

[5] Ionkova I., Shkondrov A., Krasteva I., Ionkov T. (2014). Recent progress in phytochemistry, pharmacology and biotechnology of Astragalus saponins. Phytochem. Rev..

[6] Rios J.L., Waterman P.G. (1997). A review of the pharmacology and toxicology of Astragalus. Phytother. Res..

[7] Gülcemal D., Aslanipour B., Bedir E., Ozturk M., Hakeem K.R. (2019). Secondary Metabolites from Turkish Astragalus Species. Plant and Human Health, Volume 2: Phytochemistry and Molecular Aspects.

[8] Wang Y., Auyeung K.K., Zhang X., Ko J.K. (2014). Astragalus saponins modulates colon cancer development by regulating calpain-mediated glucose-regulated protein expression. BMC Complement. Altern. Med..

[9] Yang L.P., Shen J.G., Xu W.C., Li J., Jiang J.Q. (2013). Secondary metabolites of the genus Astragalus: Structure and biological-activity update. Chem. Biodivers..

[10] Shkondrov A., Krasteva I., Bucar F., Kunert O., Kondeva-Burdina M., Ionkova I. (2018). A new tetracyclic saponin from Astragalus glycyphyllos L. and its neuroprotective and hMAO-B inhibiting activity. Nat. Prod. Res..

[11] Pistelli L., Bertoli A., Lepori E., Morelli I., Panizzi L. (2002). Antimicrobial and antifungal activity of crude extracts and isolated saponins from Astragalus verrucosus. Fitoterapia.

[12] Yin X., Zhang Y., Yu J., Zhang P., Shen J., Qiu J., Wu H., Zhu X. (2006). The antioxidative effects of astragalus saponin I protect against development of early diabetic nephropathy. J. Pharmacol. Sci..

[13] Aslanipour B., Gulcemal D., Nalbantsoy A., Yusufoglu H., Bedir E. (2017). Secondary metabolites from Astragalus karjaginii BORISS and the evaluation of their effects on cytokine release and hemolysis. Fitoterapia.

[14] Auyeung K.K., Law P.C., Ko J.K. (2014). Combined therapeutic effects of vinblastine and Astragalus saponins in human colon cancer cells and tumor xenograft via inhibition of tumor growth and proangiogenic factors. Nutr. Cancer.

[15] Debelec-Butuner B., Ozturk M.B., Tag O., Akgun I.H., Yetik-Anacak G., Bedir E., Korkmaz K.S. (2018). Cycloartane-type sapogenol derivatives inhibit NFkappaB activation as chemopreventive strategy for inflammation-induced prostate carcinogenesis. Steroids.

[16] Auyeung K.K., Han Q.B., Ko J.K. (2016). Astragalus membranaceus: A Review of its Protection Against Inflammation and Gastrointestinal Cancers. Am. J. Chin. Med..

[17] Graziani V., Scognamiglio M., Belli V., Esposito A., D’Abrosca B., Chambery A., Russo R., Panella M., Russo A., Ciardiello F. (2018). Metabolomic approach for a rapid identification of natural products with cytotoxic activity against human colorectal cancer cells. Sci. Rep..

[18] Malvezzi M., Carioli G., Bertuccio P., Rosso T., Boffetta P., Levi F., La Vecchia C., Negri E. (2016). European cancer mortality predictions for the year 2016 with focus on leukaemias. Ann. Oncol..

[19] Baselga J. (2001). The EGFR as a target for anticancer therapy - focus on cetuximab. Eur. J. Cancer.

[20] Ciardiello F., Tortora G. (2008). EGFR antagonists in cancer treatment. N. Engl. J. Med..

[21] Scognamiglio M., D’Abrosca B., Fiumano V., Chambery A., Severino V., Tsafantakis N., Pacifico S., Esposito A., Fiorentino A. (2012). Oleanane saponins from Bellis sylvestris Cyr. and evaluation of their phytotoxicity on Aegilops geniculata Roth. Phytochemistry.

[22] Wang Z.B., Zhai Y.D., Ma Z.P., Yang C.J., Pan R., Yu J.L., Wang Q.H., Yang B.Y., Kuang H.X. (2015). Triterpenoids and Flavonoids from the Leaves of Astragalus membranaceus and Their Inhibitory Effects on Nitric Oxide Production. Chem. Biodivers..

[23] Kitagawa I., Wang H., Saito M., Takagi A., Yoshikawa M. (1983). Saponin and sapogenol. XXXV. Chemical constituents of astragali radix, the root of Astragalus membranaceus Bunge. (2). Astragalosides I, II and IV, acetylastragaloside I and isoastragalosides I and II. Chem. Pharm. Bull..

[24] Tin M.M., Cho C.H., Chan K., James A.E., Ko J.K. (2007). Astragalus saponins induce growth inhibition and apoptosis in human colon cancer cells and tumor xenograft. Carcinogenesis.

[25] Auyeung K.K., Cho C.H., Ko J.K. (2009). A novel anticancer effect of Astragalus saponins: Transcriptional activation of NSAID-activated gene. Int. J. Cancer.

[26] Ionkova I., Momekov G., Proksch P. (2010). Effects of cycloartane saponins from hairy roots of Astragalus membranaceus Bge., on human tumor cell targets. Fitoterapia.

[27] Auyeung K.K., Woo P.K., Law P.C., Ko J.K. (2012). Astragalus saponins modulate cell invasiveness and angiogenesis in human gastric adenocarcinoma cells. J. Ethnopharmacol..

[28] Saif M.W. (2010). Colorectal cancer in review: The role of the EGFR pathway. Expert Opin Investig. Drugs.

[29] Veluchamy J.P., Spanholtz J., Tordoir M., Thijssen V.L., Heideman D.A., Verheul H.M., de Gruijl T.D., van der Vliet H.J. (2016). Combination of NK Cells and Cetuximab to Enhance Anti-Tumor Responses in RAS Mutant Metastatic Colorectal Cancer. PLoS ONE.

[30] Richman S.D., Seymour M.T., Chambers P., Elliott F., Daly C.L., Meade A.M., Taylor G., Barrett J.H., Quirke P. (2009). KRAS and BRAF Mutations in Advanced Colorectal Cancer Are Associated With Poor Prognosis but Do Not Preclude Benefit From Oxaliplatin or Irinotecan: Results From the MRC FOCUS Trial. J. Clin. Oncol..

[31] Roth A.D., Tejpar S., Delorenzi M., Yan P., Fiocca R., Klingbiel D., Dietrich D., Biesmans B., Bodoky G., Barone C. (2010). Prognostic role of KRAS and BRAF in stage II and III resected colon cancer: Results of the translational study on the PETACC-3, EORTC 40993, SAKK 60-00 trial. J. Clin. Oncol..

[32] Van Cutsem E., Kohne C.H., Lang I., Folprecht G., Nowacki M.P., Cascinu S., Shchepotin I., Maurel J., Cunningham D., Tejpar S. (2011). Cetuximab plus irinotecan, fluorouracil, and leucovorin as first-line treatment for metastatic colorectal cancer: Updated analysis of overall survival according to tumor KRAS and BRAF mutation status. J. Clin. Oncol..

[33] Chapman P.B., Hauschild A., Robert C., Haanen J.B., Ascierto P., Larkin J., Dummer R., Garbe C., Testori A., Maio M. (2011). Improved survival with vemurafenib in melanoma with BRAF V600E mutation. N. Engl. J. Med..

[34] Kopetz S., Desai J., Chan E., Hecht J.R., O’Dwyer P.J., Lee R.J., Nolop K.B., Saltz L. (2010). PLX4032 in metastatic colorectal cancer patients with mutant BRAF tumors. J. Clin. Oncol..

[35] Prahallad A., Sun C., Huang S.D., Di Nicolantonio F., Salazar R., Zecchin D., Beijersbergen R.L., Bardelli A., Bernards R. (2012). Unresponsiveness of colon cancer to BRAF(V600E) inhibition through feedback activation of EGFR. Nature.

[36] Scognamiglio M., Fiumano V., D’Abrosca B., Esposito A., Choi Y.H., Verpoorte R., Fiorentino A. (2014). Chemical interactions between plants in Mediterranean vegetation: The influence of selected plant extracts on Aegilops geniculata metabolome. Phytochemistry.

