# Supplementary material for: Spectroscopic Characterization and Cytotoxicity Assessment towards Human Colon Cancer Cell Lines of Acylated Cycloartane Glycosides from Astragalus boeticus L
Source: Molecules. 2019 May 3;24(9):1725. doi: 10.3390/molecules24091725 (PMC6539726; doi:10.3390/molecules24091725)
Supplement: Supplementary file 1 [file molecules-24-01725-s001.pdf]

Type of the Paper (Article)

Spectroscopic Characterization and Cytotoxicity Assessment towards Human Colon Cancer Cell Lines of Acylated cycloartane glycosides from *Astragalus boeticus* L.

Vittoria Graziani[1], Assunta Esposito[1], Monica Scognamiglio[2,*], Angela Chambery[1], Rosita Russo[1], Fortunato Ciardiello[3], Teresa Troiani[3], Nicoletta Potenza[1], Antonio Fiorentino[1,4,*], Brigida D'Abrosca[1,4]

[1] Dipartimento di Scienze e Tecnologie Ambientali Biologiche e Farmaceutiche (DiSTABiF), Università degli Studi della Campania "Luigi Vanvitelli", via Vivaldi 43 I-81100, Caserta, Italy; vittoria.graziani@unicampania.it (V.G.); assunta.esposito@unicampania.it (A.S.); angela.chambery@unicampania.it (A.C.); rosita.russo@unicampania.it (R.R.); nicoletta.potenza@unicampania.it (N.P.); antonio.fiorentino@unicampani.it (A.F.); brigida.dabrosca@unicampania.it (B.D.A)

[2] Department of Biochemistry, Max Planck Institute for Chemical Ecology - Beutenberg Campus, Hans-Knöll-Straße 8 D-07745 Jena, Germany; mscognamiglio@ice.mpg.de

[3] Dipartimento di Medicina di Precisione, Università degli Studi della Campania "Luigi Vanvitelli" - Via Pansini, 5, 80131, Napoli, Italy; fortunato.ciardiello@unicampania.it (F.C.); teresa.troiani@unicampania.it (T.T.)

[4] Dipartimento di Biotecnologia Marina, Stazione Zoologica Anton Dohrn, Villa Comunale, Naples 80121, Italy

*Correspondence: mscognamiglio@ice.mpg.de (M.S.), antonio.fiorentino@unicampania.it (A.F.); Tel.: +49 (0) 3641 5701609 (M.S.), +39 (0) 823 274576 (A.F.)

Supplementary material

Figure S1: ^{1}H NMR spectrum of compound **1**

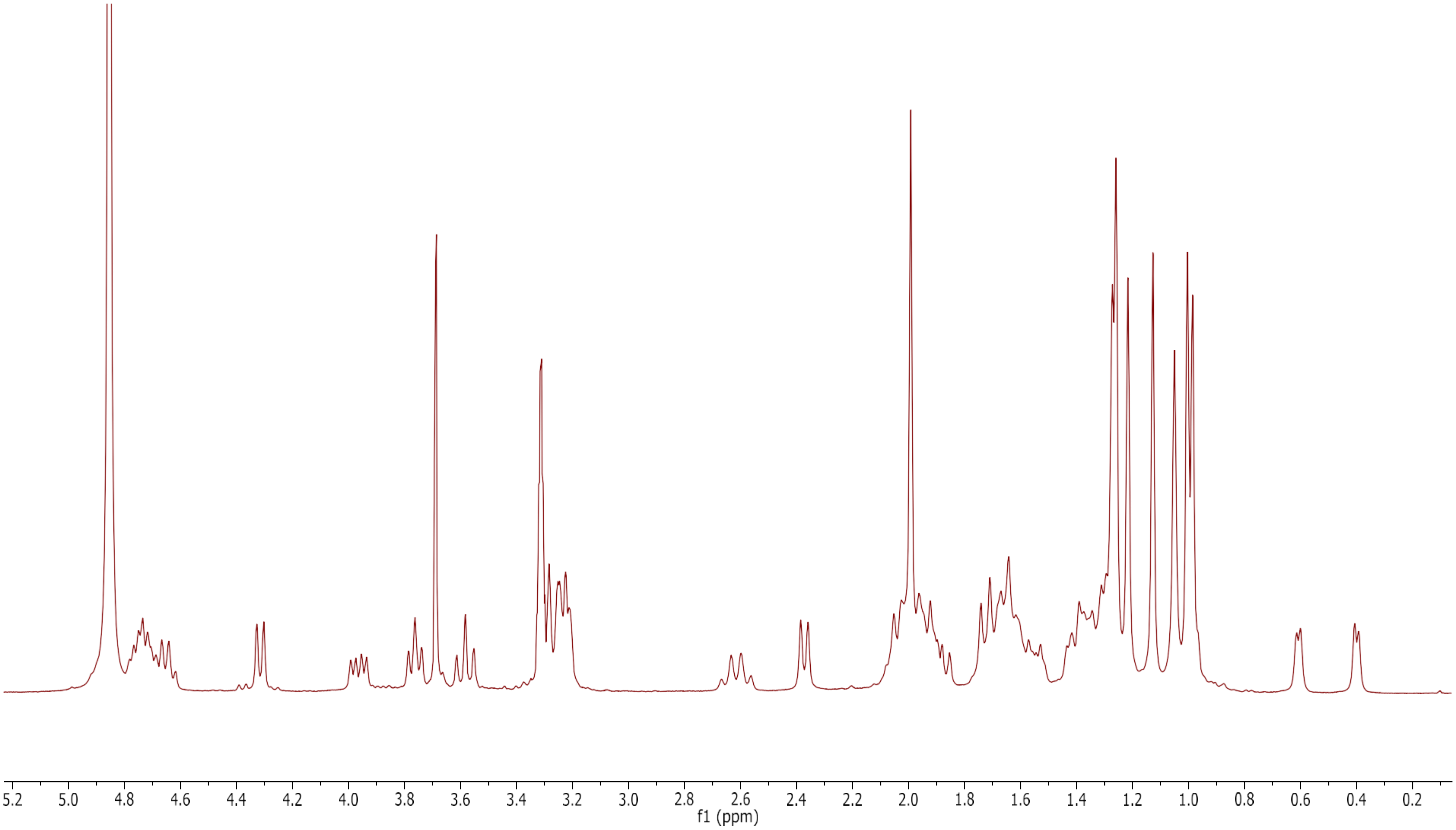

Figure S2: ^{13}C NMR spectrum of compound **1**

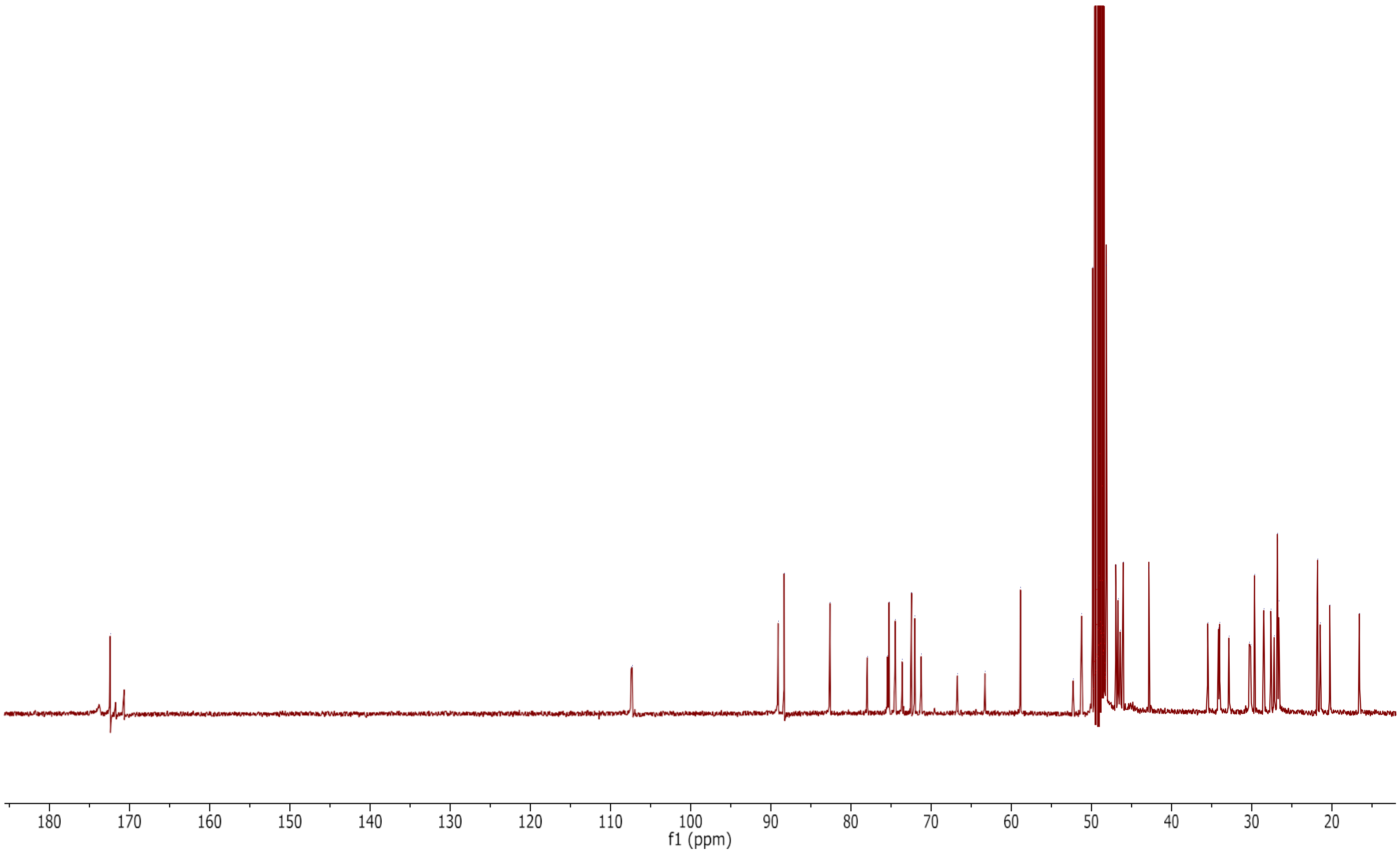

Figure S3: COSY spectrum of compound **1**

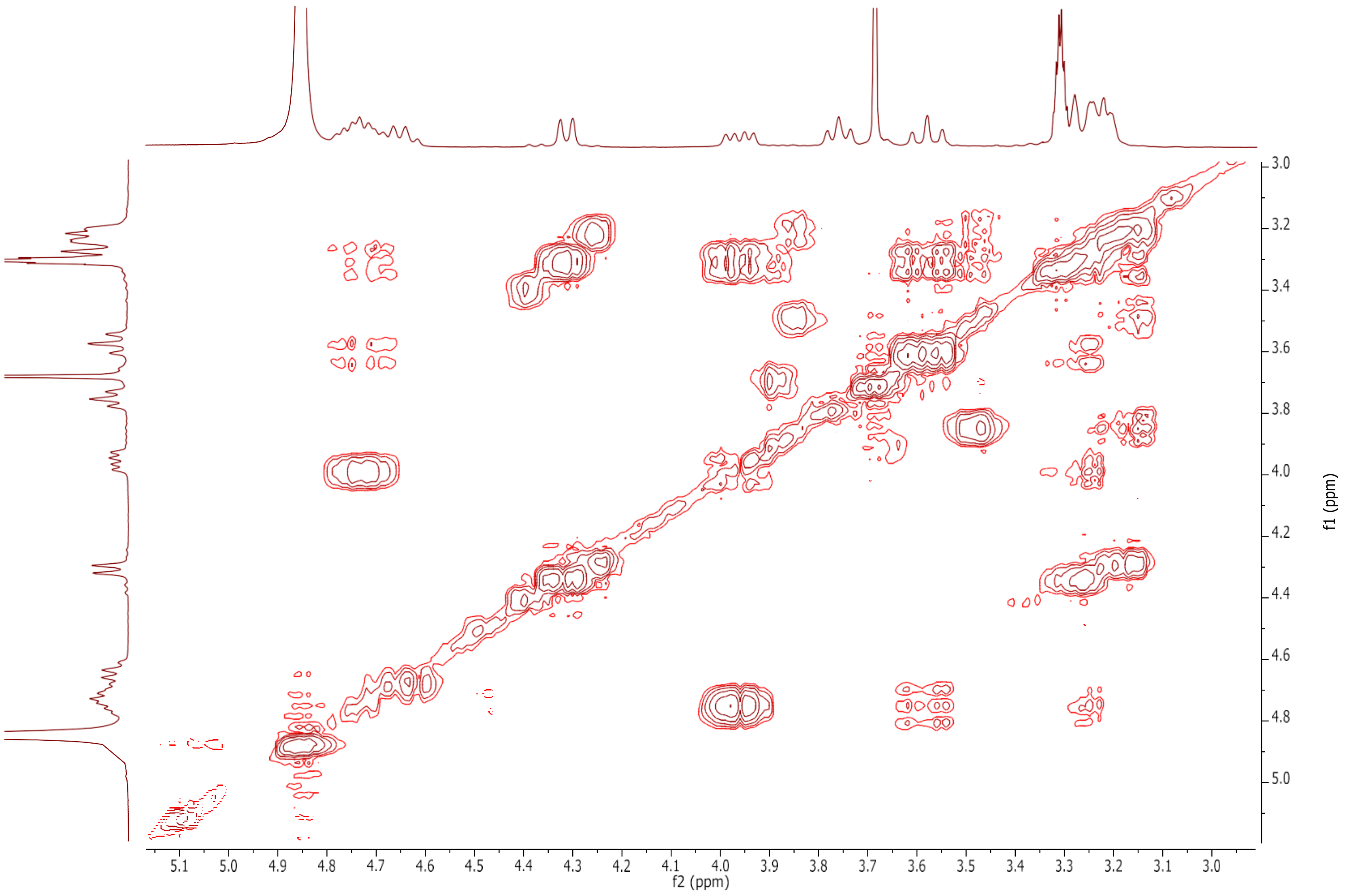

Figure S4: HSQC spectrum of compound **1**

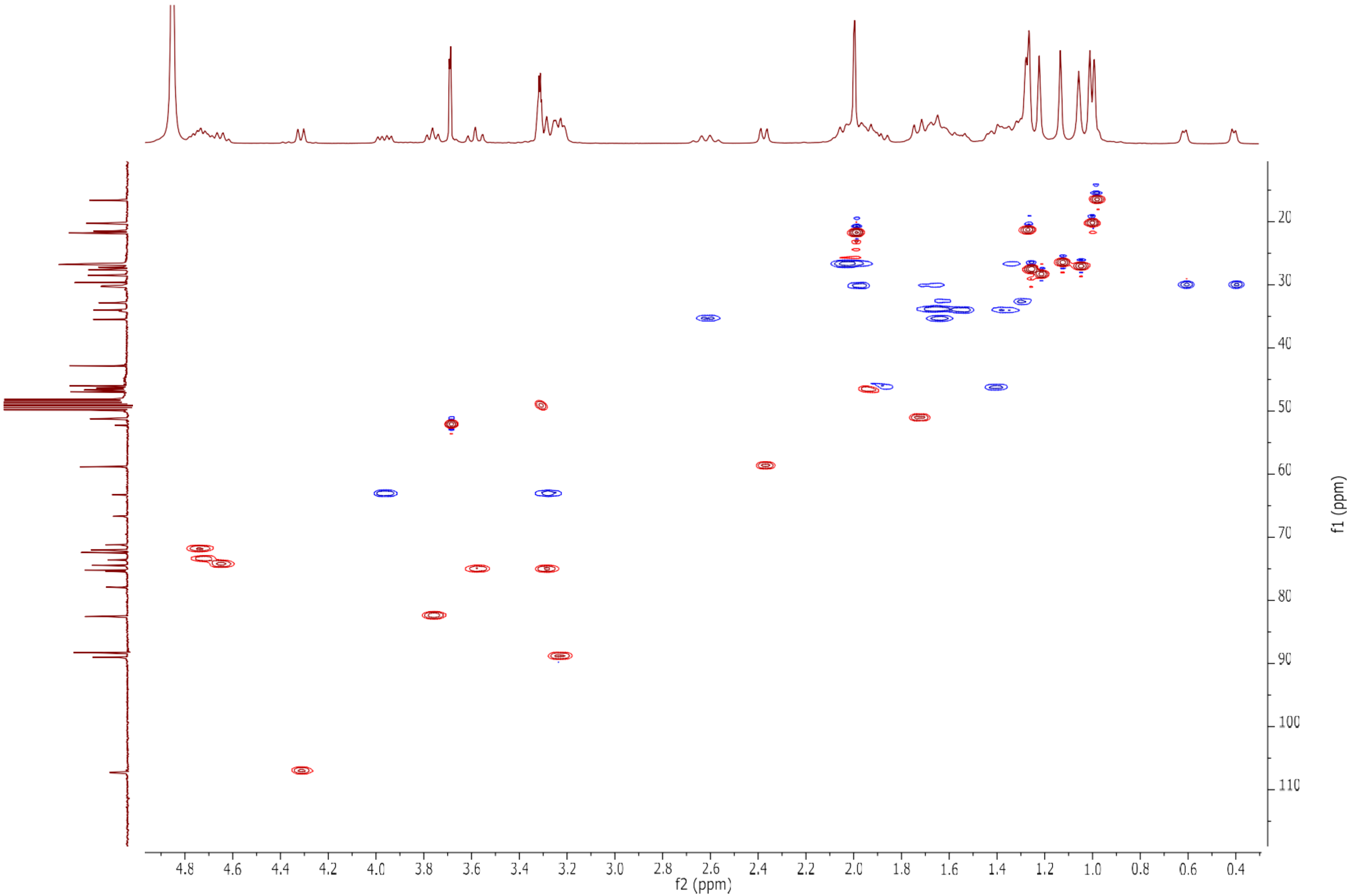

Figure S5: CIGAR-HMBC spectrum of compound **1**

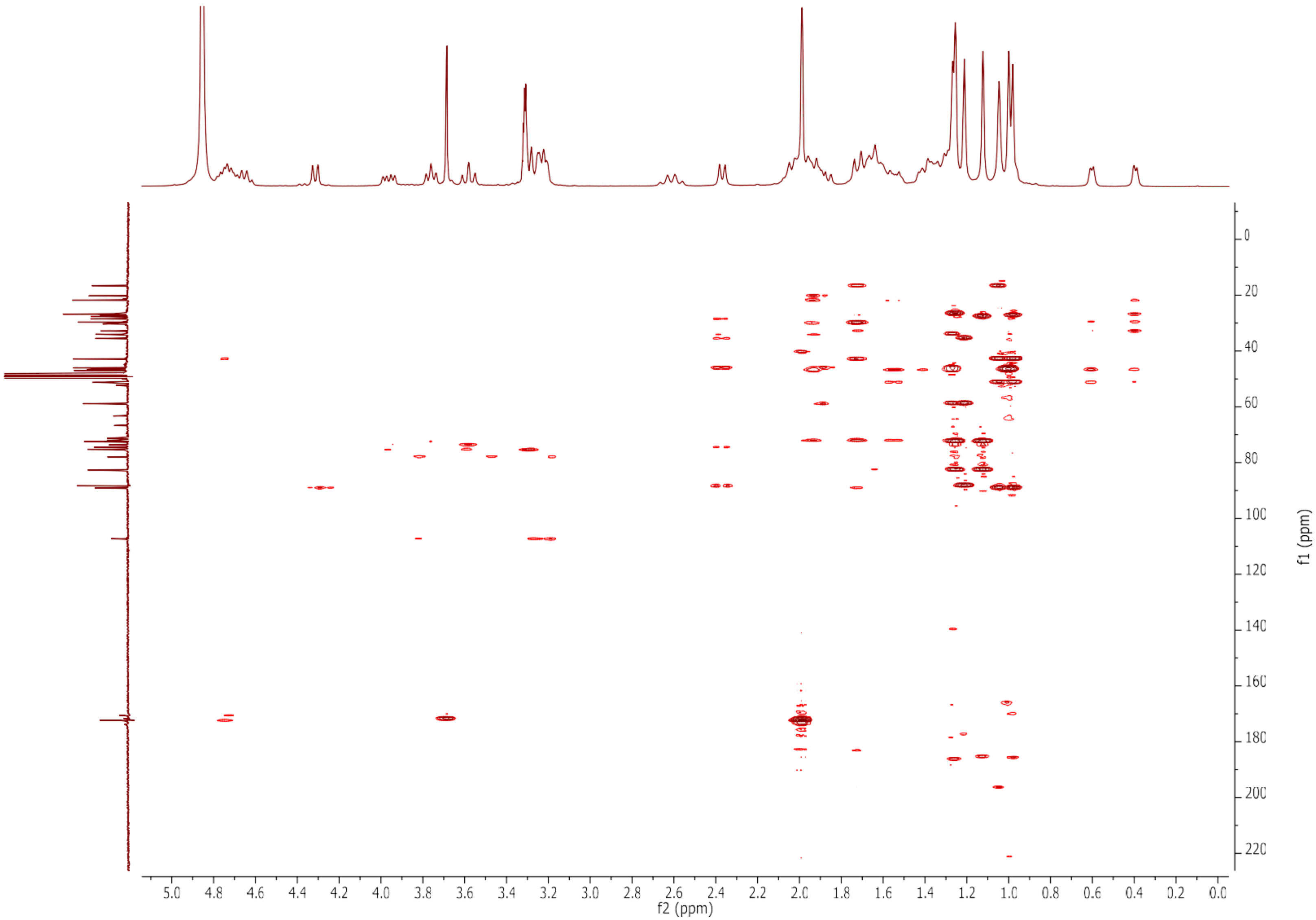

Figure S6: H2BC spectrum of compound **1**

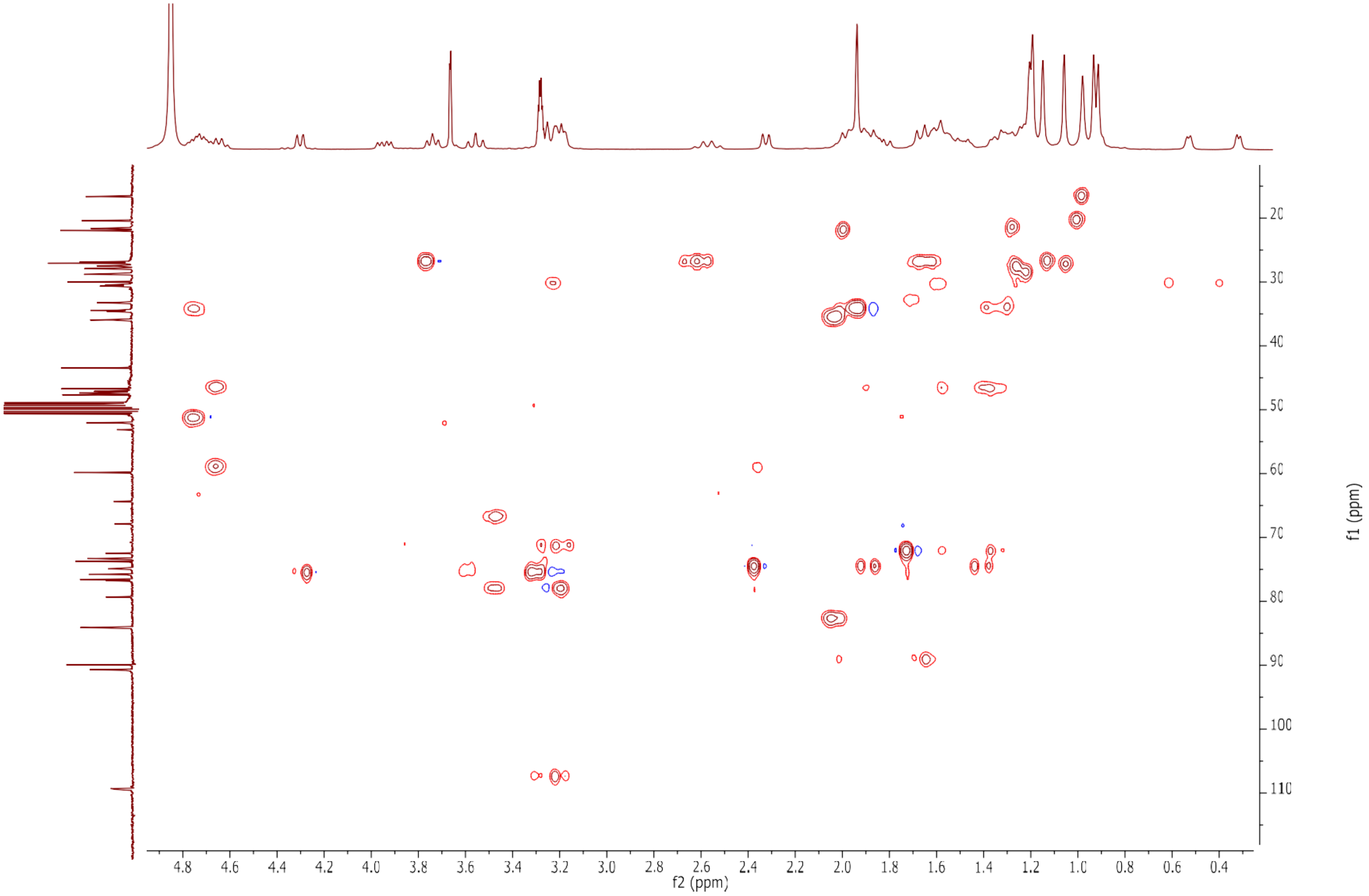

Figure S7: HSQCTOCSY spectrum of compound **1**

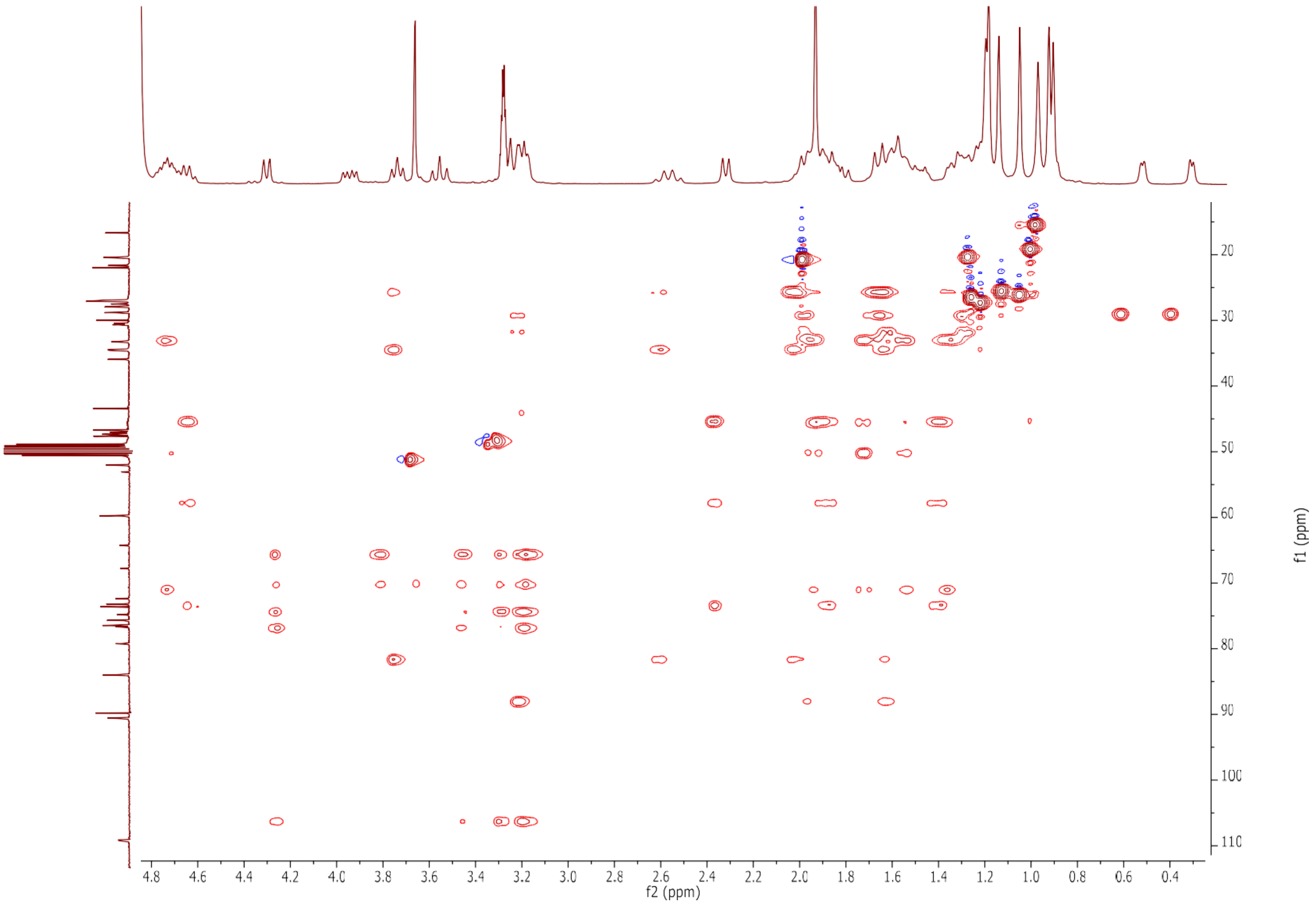

Figure S8: ESI QTOF spectrum of compound **1**

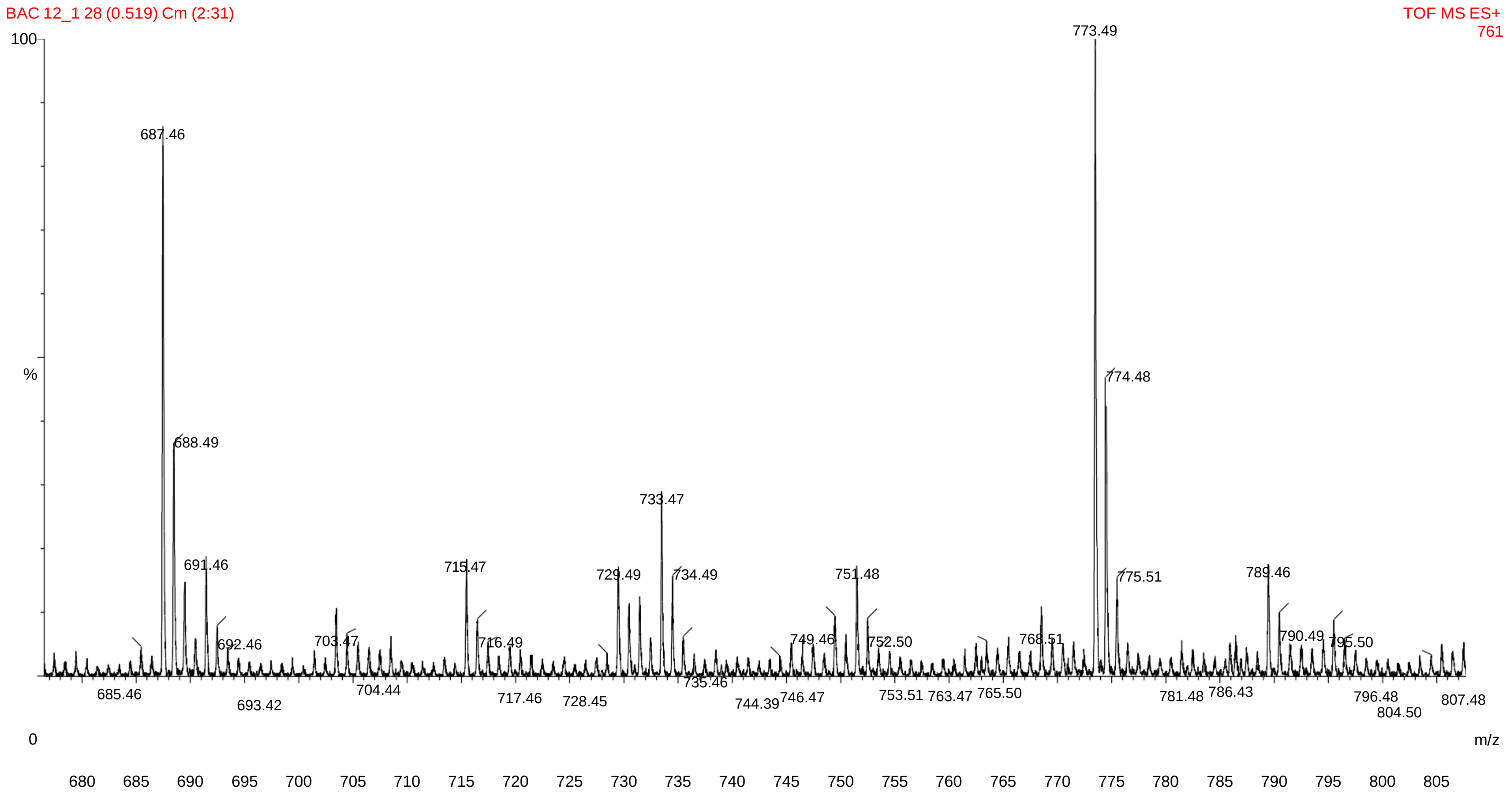

Figure S9: ESI TOF MS/MS spectrum of compound **1**

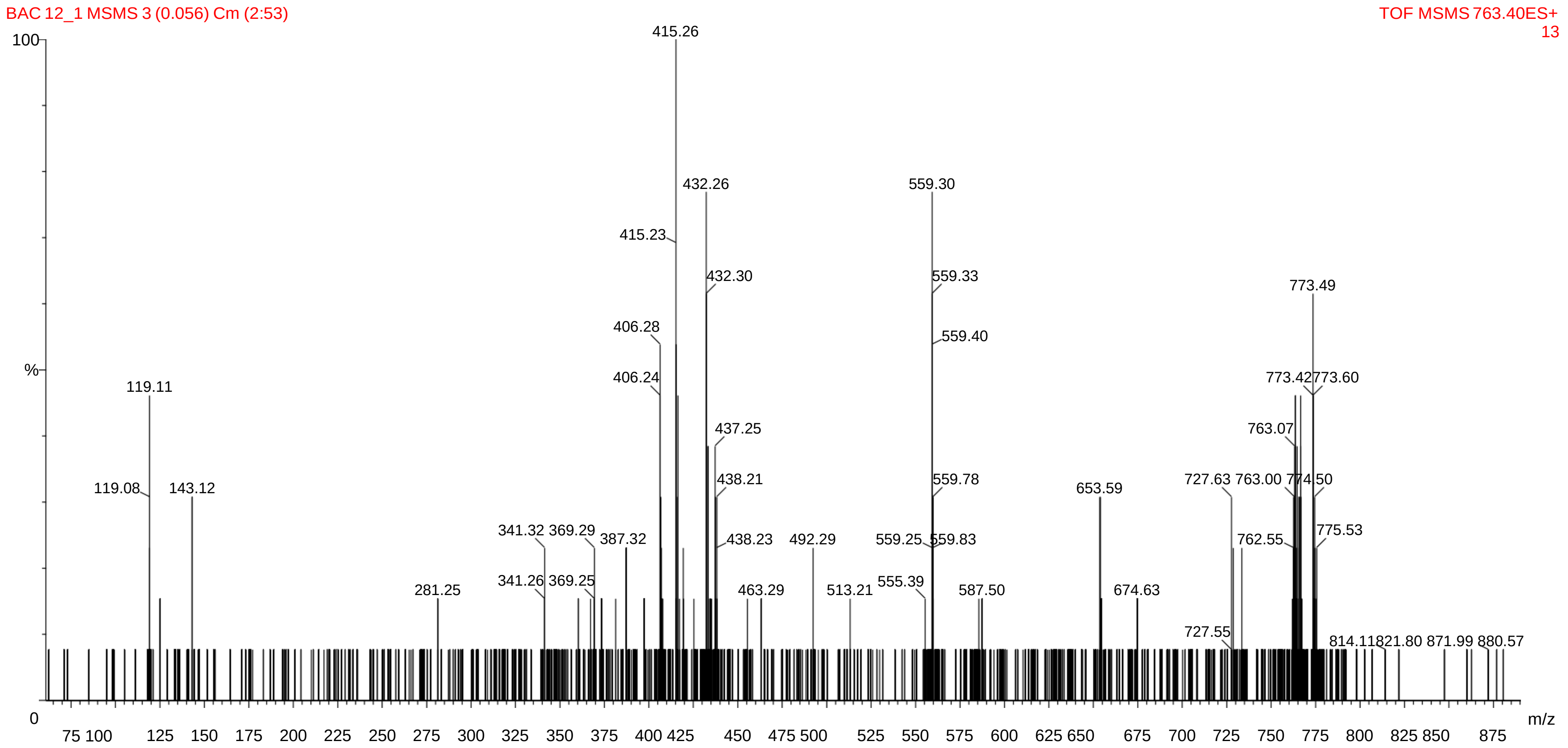

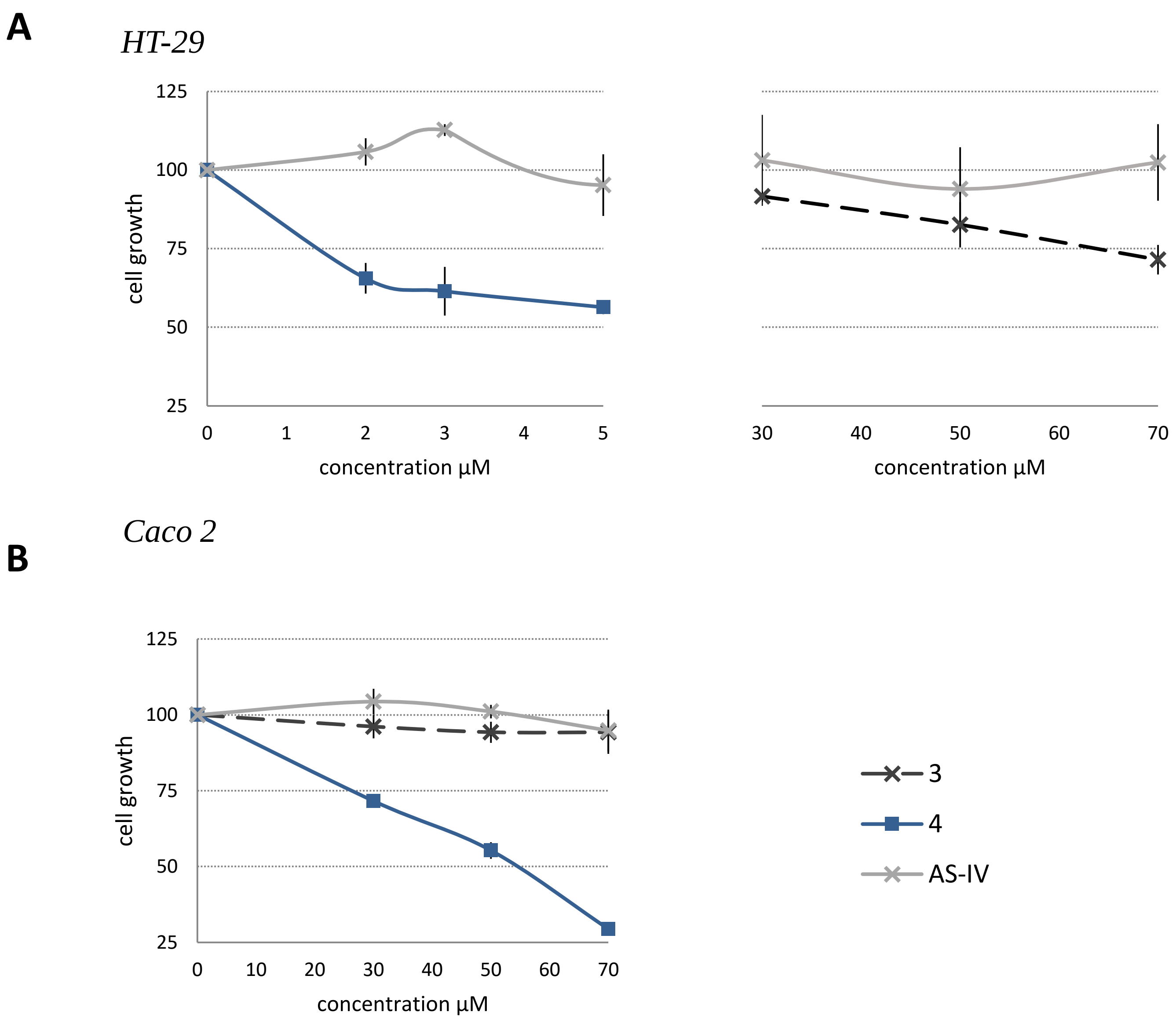

Figure S10: cytotoxicity of AS-IV in comparison with compound **3** and **4**

A Cytotoxic activity of AS-IV and the active compound **4** at 2, 3 and 5 µM (graph on the left) in HT-29 cell line. Cytotoxic activity of AS-IV and the inactive compound **3** at 30, 50 and 70 µM (graph on the right) in HT-29 cell line.
B Cytotoxic activity of AS-IV, compound **3** and **4** at 30, 50 and 70 µM in Caco-2 cell line (graph on the left). Figure legend on the right.
